# Diving into the CO_*x*_ Methanation Mechanism of CO and CO_2_ Mixtures Catalyzed by the Nanostructured NiO-CeO_2_ Catalyst

**DOI:** 10.1021/acscatal.5c02682

**Published:** 2025-07-05

**Authors:** Iván Martínez-López, Juan Bueno-Ferrer, Iris Martín-García, Esteban Guillén-Bas, Arantxa Davó-Quiñonero, Virginia Pérez-Dieste, Dolores Lozano-Castelló, Agustín Bueno-López

**Affiliations:** † Department of Inorganic Chemistry, 16718 University of Alicante, Carretera de San Vicente s/n, 03690 Alicante, Spain; ‡ ALBA Synchrotron Light Source, Carrer de la Llum 2-26, 08290 Cerdanyola del Vallès, Barcelona, Spain

**Keywords:** CO_*x*_ methanation, nickel, ceria, in situ DRIFTS, isotopic experiments, NAP-XPS, reaction mechanism

## Abstract

The CO_2_ methanation process in the presence of CO is a catalytic challenge toward the abatement of CO_2_ emissions from industrial exhaust gases with the goal of producing CH_4_ as a clean fuel. Herein, the CO_*x*_ methanation mechanisms of a NiO-CeO_2_ (Np) catalyst constituted by nickel oxide-cerium oxide nanoparticles with efficient operation under CO_2_ and CO + CO_2_ gas mixtures were addressed. In situ diffuse reflectance infrared Fourier-transform spectroscopy (DRIFTS) analyses and ^13^C^18^O_2_ (49) pulse isotopic experiments reveal that solo CO_2_ methanation and comethanation (CO + CO_2_) present a common mechanism in which CO_2_ is transformed into *CO, from where the reaction proceeds. According to our outputs, inlet CO interferes with the CO_2_ methanation activity, delaying the reaction onset without selectivity impacts. Conversely, in the absence of CO_2_, the solo CO methanation performance is remarkably limited with poor CH_4_ selectivity. As demonstrated, the solo CO methanation mechanism is based on formates as key intermediates at low temperatures (<250 °C), while competitive CO_2_ production takes place onward. Near-ambient pressure X-ray photoelectron spectroscopy (NAP-XPS) analyses conducted in synchrotron revealed a higher degree of reduction in the catalyst when CO and H_2_ are fed either alone or in combination with CO_2_, attributed to the CO oxygen abstraction capacity from the CeO_2_ phase. In the comethanation mixture, CO_2_ and CO are balanced in oxidation–reduction processes, yielding the maximum CO_*x*_ conversion, while in the solo CO methanation, reductive processes prevail, limiting the CH_4_ formation.

## Graphical abstract

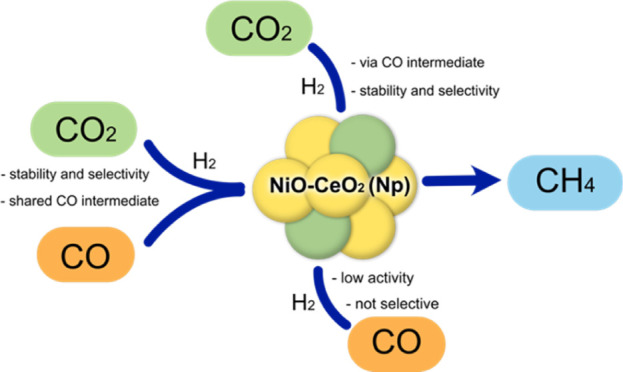

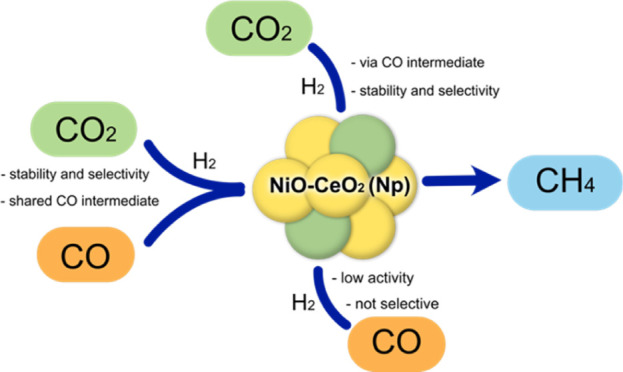

## 1 Introduction

The hydrogenation of carbon oxides (CO_*x*_) into methane is attracting interest, driven by the growing environmental awareness and the evolving energy policies aiming at the reduction of greenhouse gas emissions aiming the mitigation of global warming.^1–3^ In the *power-to-ga*s approach, CO_*x*_ (variable mixtures of CO and CO_2_) streams captured from conventional fossil-fuel power plants' flue gas, from biomass transformation, or from coal gasification plants, are converted into synthetic natural gas (SNG) using green H_2_ produced by renewable energy. This reaction system, involving CO, CO_2_, H_2_O, CH_4_, and H_2_, is governed by a network of reactions, primarily the methanation of CO and CO_2_ (eq 1 and 2, respectively).

$$\begin{array}{cc} \mathrm{CO}+3H_{2}⇌{\mathrm{CH}}_{4}+H_{2}O & \Delta {H^{0}}_{298K}=-206\;\mathrm{kJ}/\mathrm{mol} \end{array}$$ 1

$$\begin{array}{cc} {\mathrm{CO}}_{2}+4H_{2}⇌{\mathrm{CH}}_{4}+2H_{2}O & \Delta {H^{0}}_{298K}=-165\;\mathrm{kJ}/\mathrm{mol} \end{array}$$ 2

Feed gases from coal gasification or biomass plants typically contain CO, which significantly inhibits CO_2_ methanation.^4,5^ Furthermore, elevated CO concentrations can cause severe catalyst deactivation, either through carbon deposition or by forming volatile Ni­(CO)_4_ compounds.^6,7^

While Ru is the most active metal, its limited availability and high cost hinder the large-scale commercialization of Ru-based catalysts. In contrast, Ni-based systems are more appealing for CO_*x*_ methanation to SNG due to their low cost and high selectivity toward methane.^8,9^ Active Ni catalysts supported on CeO_2_ phases have demonstrated good CO_2_ and CO conversions and excellent selectivity toward CH_4_.^10–13^ As reported previously in our group, NiO-CeO_2_ (Np) catalysts based on nanoparticles exhibited an encouraging operation without forming coke or irreversible carbonyl species in methane dry reforming. Furthermore, the catalyst presented an excellent efficiency in the CO_2_ methanation reaction with high selectivity toward CH_4_.^14,15^

Nowadays, for both CO and CO_2_ methanation, two distinct mechanisms have been proposed–one involving an intact intermediate and another involving bond cleavage–based on the state of the carbon-containing surface species before hydrogenation. In the pathway where intermediates remain intact for CO_2_ methanation, CO_2_ is initially hydrogenated into formate, which then undergoes hydrogenation to yield CH_4_.^6,16,17^

In the other mechanism, involving bond cleavage, the CO_2_ molecules attach dissociatively to the catalyst surface, forming a CO* intermediate. This intermediate undergoes stepwise hydrogenation, creating partially hydrogenated species, which continue reacting with hydrogen as the process advances.^18,19^ Alternatively, the CO* intermediate could further break into a C* species, which then undergoes sequential hydrogenation to produce CH_4_.^20^ Investigations on metal–supported catalysts have revealed that CO and CO_2_ methanation could share a common mechanism, involving similar intermediates and reaction pathways.^21–23^ During methanation, CO_2_ initially interacts with the oxidic phases of the catalyst, forming carbonates that are subsequently hydrogenated into formate. Formates decompose into CO* on the metal sites, which serves as a primary intermediate to produce both CH_4_ and CO.^22^ In contrast, CO directly binds to the metal centers, bypassing the carbonate and formate intermediates.^19^

The stronger binding affinity of CO to active metal sites and its faster adsorption compared to CO_2_ often leads to an inhibitory effect on CO_2_ methanation.^21^ When CO is present, CO_2_ adsorption can become the rate-limiting step, slowing its methanation activity. Additionally, factors that promote CO methanation while inhibiting CO_2_ methanation in hydrocarbon reformates have been elucidated. These include the particle size and electronic state of the active species, as well as the CO_2_ adsorption properties and surface characteristics of the catalyst supports.^24^ These findings suggest that while both CO and CO_2_ methanation follow a shared sequence of steps, their interactions with the catalyst differ, influencing the overall efficiency and selectivity of the process.

In this work, we focus on the mechanism of simultaneous methanation of CO and CO_2_ catalyzed by NiO-CeO_2_ (Np) nanoparticles. The experimental outputs on the mechanism assessed herein describe how CO methanation is enhanced by CO_2_ copresence. Thus, CO and CO_2_ methanation in the comethanation mixture present mechanisms with a shared kinetic route once CO_2_ is transformed into CO in the first steps. Moreover, the great stability of the NiO-CeO_2_ (Np) catalyst in the simultaneous methanation of CO and CO_2_ has been demonstrated by NAP-XPS.

## 2 Experimental Details

### 2.1 Catalysts Preparation

The metal precursors of Ce and Ni for the catalyst preparation were Ce­(NO_3_)_3_·6H_2_O (99.5%, Alfa Aesar), Ni­(NO_3_)_2_·6H_2_O (Panreac), and NiO-CeO_2_ (Np). The reverse microemulsion method was used to synthesize NiO-CeO_2_ nanoparticles.^25^ Two separate microemulsions were prepared. The first microemulsion included the metal precursors, 1.18 g of Ni precursor and 4.27 g of Ce precursor dissolved in 20 mL of water, which was then mixed with an organic phase consisting of 167 mL of *N*-heptane, 34 mL of Triton X-100 (surfactant), and 35 mL of hexanol (cosurfactant). In the second microemulsion, the same amounts of *N*-heptane, Triton X-100, and hexanol were used, but instead of metal precursors, 6.8 g of tetramethylammonium hydroxide pentahydrate was added. The two microemulsions were subsequently combined and stirred for 24 h. Afterward, the precipitate was isolated by centrifugation, rinsed with ethanol, dried at 110 °C, and calcined at 500 °C for 1 h. The resultant NiO-CeO_2_ (Np) catalyst is constituted by uniform 7 nm-sized binary nanoparticles where the nickel oxide phase is finely distributed within the ceria matrix. The complete catalyst characterization was previously reported in our published studies, finding a developed microporous-mesoporous surface, enhanced nickel and cerium redox synergism, and a high CO_2_ adsorption capacity in the form of weak carbonates and bicarbonates.^15,25,26^

### 2.2 CO_2_, CO, and Simultaneous CO + CO_2_ Methanation Tests

The catalytic tests were evaluated in the CO, CO_2_, and simultaneous CO + CO_2_ methanation reaction. A stainless-steel cylindrical reactor was used (0.82 cm inner diameter). The outlet gas composition was monitored by a gas chromatograph (Agilent 8860 GC System) packed with two columns: a Porapak Q 80/100 for CO_2_ separation; and a Molecular Sieve 13X for O_2_, N_2_, CH_4_, and CO separation, coupled to a TCD detector for the outlet gases analysis.

A total of 200 mg of NiO-CeO_2_ (Np) was reduced in situ before the experiments at 400 °C for 1 h under 200 mL/min of 50% H_2_/N_2_.

Catalytic tests for CO_2_ methanation were conducted from 200 to 400 °C under 200 mL/min of 10% CO_2_, 40% H_2_, and 50% N_2_.

Catalytic tests for CO methanation were carried out with 200 mL/min of 1.5% CO, 13.5% H_2_ and N_2_ balance.

Catalytic tests for CO + CO_2_ methanation were performed from 200 to 400 °C under 200 mL/min of 10% CO_2_, 1,5% CO, 50% H_2_, and N_2_ balance.

Lectures on the gas composition were taken at different temperatures after stabilization for 30 min. CO_*x*_ conversion (*X*_CO*x*_) and CH_4_ selectivity (*S*_CH4_) parameters were calculated according to the following equations:

$$X_{{\mathrm{CO}}_{2}}=\frac{[{\mathrm{CO}}_{2}{]}_{\mathrm{in}}-[{\mathrm{CO}}_{2}{]}_{\mathrm{out}}}{[{\mathrm{CO}}_{2}{]}_{\mathrm{in}}}\times 100$$ 3

$$X_{\mathrm{CO}}=\frac{[\mathrm{CO}{]}_{\mathrm{in}}-[\mathrm{CO}{]}_{\mathrm{out}}}{[\mathrm{CO}{]}_{\mathrm{in}}}\times 100$$ 4

$$S_{{\mathrm{CH}}_{4}}(A)=\frac{\left[{\mathrm{CH}}_{4}\right]}{\left[{\mathrm{CH}}_{4}]+[\mathrm{CO}\right]}\times 100$$ 5

$$S_{{\mathrm{CH}}_{4}}(B)=\frac{\left[{\mathrm{CH}}_{4}\right]}{\sum [{\mathrm{CO}}_{x}{]}_{\mathrm{in}}-[{\mathrm{CO}}_{x}{]}_{\mathrm{out}}}\times 100$$ 6

$$S_{{\mathrm{CH}}_{4}}(C)=\frac{\left[{\mathrm{CH}}_{4}\right]}{\left[{\mathrm{CH}}_{4}]+[{\mathrm{CO}}_{2}\right]}\times 100$$ 7

The CH_4_ selectivity formula was adapted to the specific conditions of each experiment, eq 5 being applied to CO_2_ methanation, eq 6 to comethanation, and eq 7 to CO methanation.

### 2.3 NAP-XPS Experiments

Near-ambient pressure (NAP) XPS experiments on the NiO-CeO_2_ (Np) catalyst were conducted at a synchrotron light source, utilizing a beamline equipped for tunable photon energies ranging from 100 to 2000 eV. A PHOIBOS NAP150 energy analyzer (SPECS GmbH) was used to collect data, with the beam focused to a spot size of 100 × 20 μm^2^ and achieving an energy resolution better than 0.3 eV. In this study, XPS spectra were recorded with a photon energy of 1090 eV, corresponding to an inelastic mean free path (IMFP) of approximately 1.9 nm for CeO_2_. According to X-ray diffraction (XRD) analysis, the primary ceria crystals in NiO-CeO_2_ exhibit an average size of 6–7 nm.^25^ The catalyst was pelletized using a gold mesh to prevent surface charging, with the experimental verification confirming that the mesh did not contribute to catalytic activity. Binding energies were corrected using the well-resolved *u‴* peak of the Ce 3d_3/2_ region assigned to Ce^4+^ cations (always present) with center at 916.9 eV.

The temperature was monitored using a K-type thermocouple, and the reaction chamber pressure was kept steady at 1 mbar throughout the experiment, with a fixed total gas flow rate of 15 mL/min.

The experimental protocol for the NAP-XPS experiments is illustrated in Scheme 1. In H_2_, CO_2_, and CO steps, 15 mL/min of the pure gases were fed. On the other hand, H_2_ + CO_*x*_ steps were fed with the stoichiometric mixtures employed in the catalytic tests.

**Scheme 1:**
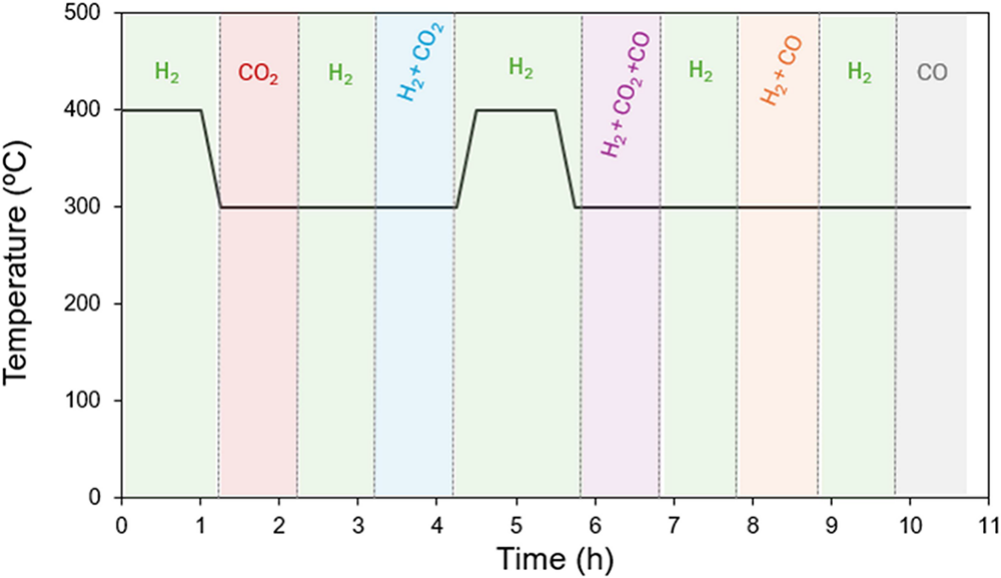
Scheme of the Protocol of Experiments in Synchrotron

### 2.4 In Situ DRIFT-MS and Isotopic Gas Pulse Experiment

In situ DRIFTS-MS experiments and isotopic gas pulse experiments were carried out in a PerkinElmer spectrometer, model Spectrum 3, using a Praying Mantis DRIFT (diffuse reflectance infrared Fourier transform spectroscopy) reaction camera coupled to a Pfeiffer vacuum mass spectrometer, model QMG 250 M1.

Two types of experiments were carried out in this experimental set up, including experiments under different gas flows and pulse experiments. In experiments performed under different gas flows, the catalyst (200 mg) was first reduced in situ at 400 °C for 1 h under 50% H_2_/N_2_, and then the temperature was decreased in N_2_ to 300 °C. The background was subtracted in these conditions. Subsequently, the reactor was fed with 50 mL/min, 10% CO_2_ + 40% H_2_ + N_2_ or 1.5% CO, 13.5% H_2_ or 10% CO_2_, 1.5% CO, and 50% H_2_ in N_2_ (balance), and the gas composition was successively switched between the stream gases and atmospheres that only include one of the reactants (40% H_2_/N_2_, 10% CO_2_/N_2_, and 1.5% CO/N_2_). Spectra were recorded after 30 min stabilization under each of the conditions. Outlet gases from the DRIFTS high-temperature chamber were monitored by mass spectrometry. No products other than CH_4_ and CO were detected under any of the tested conditions.

Other DRIFT experiments were carried out to study the mechanisms with temperature. The catalyst was first in situ reduced at 400 °C for 1 h under 50% H_2_/N_2_, and then the temperature was decreased in N_2_ to 200 °C. The background was recorded in these conditions. Subsequently, the reactor was fed with 100 mL/min, 1.5% CO, 13.5% H_2_ in N_2_ (balance) or 10% CO_2_, 1.5% CO, 50% H_2_ in N_2_ (balance) and the temperature increased up to 350 °C, recording spectra at 200, 250, 300, and 350 °C after 30 min stabilization.

On the other hand, isotopic ^13^C^18^O_2_ (*m*/*Q* = 49) and ^12^C^18^O (*m*/*Q* = 30) pulse experiments were also carried out with the NiO-CeO_2_ (Np) catalyst at different temperatures (200, 250, 300, and 350 °C). The catalyst was reduced in situ at 400 °C for 15 min under 50% H_2_/He, and then the temperature was decreased and stabilized in 50% H_2_/He at the desired temperature. Then, a pulse of isotopic ^13^C^18^O_2_ or ^12^C^18^O (0.53 mL and 0.8 bar) was fed to the H_2_/He gas stream, and the gas products were monitored by mass spectrometry. At the same time, the surface of the catalyst was monitored by fast-screening infrared spectroscopy, recording 60 spectra per minute. Then, the catalyst was cleaned and reduced again in situ at 400 °C for 15 min under 50% H_2_/He, and the protocol of the pulse experiments was repeated with different temperatures.

A background spectrum was recorded under 50% H_2_/He before each temperature for all the experiments except for the ^12^C^18^O over methanation atmosphere; for this pulse, the background recorded was CO_2_/H_2_ at each temperature.

## 3 Results and Discussion

### 3.1 CO_*x*_ Methanation Activity Tests

#### 3.1.1 Solo CO_2_ Methanation (CO_2_ + H_2_)

The solo CO_2_ methanation reaction (Figure 1) catalyzed by the NiO-CeO_2_ (Np) catalyst presents its onset around 190 °C, with its maximum CO_2_ conversion (*X*_CO2_) of 86% achieved at 350 °C and limited by thermodynamics. As depicted, the catalyst presents excellent selectivity toward CH_4_ with values near 100% in the whole range of temperatures tested. This means that no CO is released in these experiments, the CO_2_ reduction being fully selective to the formation of CH_4_. In previous studies, we have reported the excellent stability and cyclability of the NiO-CeO_2_ (Np) catalyst in the CO_2_ methanation, also confirmed by the 10-h isothermal test displayed in Figure 1b.^15^

**Figure 1:**
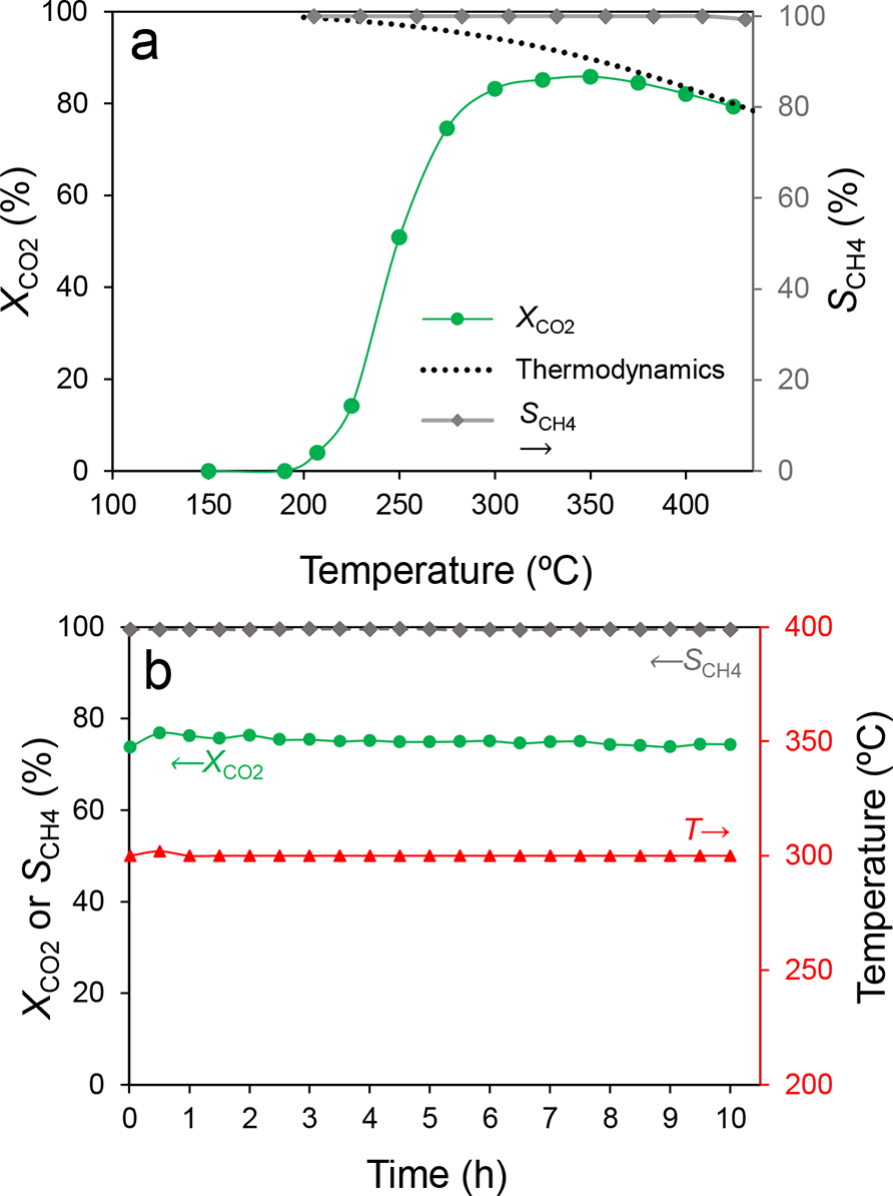
(a) CO_2_ conversion (*X*_CO2_) profile (solid line) and CH_4_ selectivity (*S*_CH4_, dashed line) in the fixed-bed activity test in the solo CO_2_ methanation with the NiO-CeO_2_ (Np) catalyst. (b) 10 h isothermal (300 °C) CO_2_ methanation test.

Thus, the NiO-CeO_2_ (Np) catalyst is active, stable, and selective toward methane in the CO_2_ + H_2_ experimental conditions.

#### 3.1.2 Co-Methanation (CO_2_ + CO + H_2_)

The comethanation activity test, including CO_2_ and CO in the feeding mixture (Figure 2) reveals that CO methanation begins at a lower temperature when compared to CO_2_ methanation in the same conditions (200 °C), reaching a 98% conversion at 300 °C. Meanwhile, CO_2_ methanation is negatively affected by the copresence of CO, with its onset shifted 50 °C when compared to solo CO_2_ methanation. However, beyond 300 °C, CO_2_ conversion profiles in the comethanation match those of the standalone CO_2_ methanation (Figure 1a), which implies that CO adsorption on the catalyst active sites affects CO_2_ activation on the catalyst surface. Thus, once CO is hydrogenated at a significant rate and its coverage on the catalyst surface is a minimum, CO_2_ methanation proceeds. The interrelated roles of CO and CO_2_ in comethanation when both are cofed to the reactor will be discussed later.

**Figure 2:**
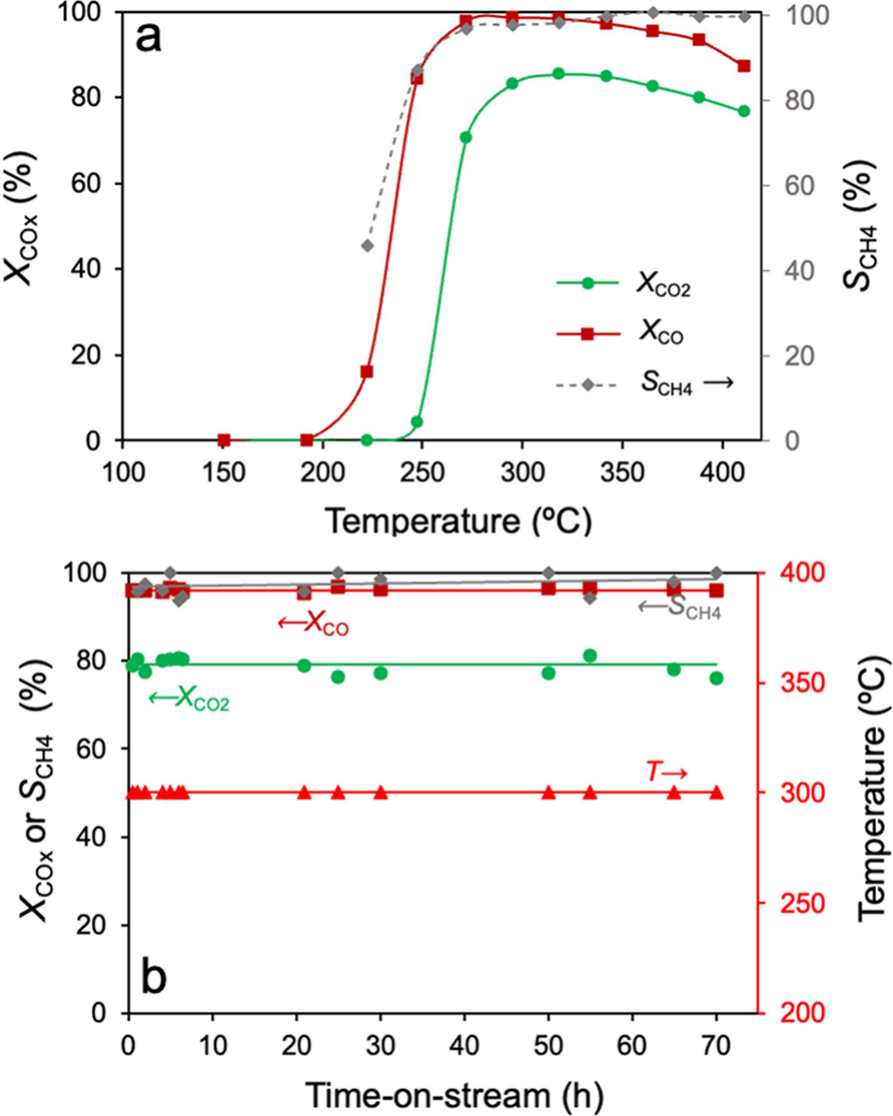
(a) CO_2_ conversion profile (*X*_CO2_, circles), CO conversion (*X*_CO_, squares) and CH_4_ selectivity (*S*_CH4_, dashed line) in the fixed-bed activity test in the comethanation mixture with the NiO-CeO_2_ (Np) catalyst. (b) 70 h isothermal (300 °C) comethanation test.

The stability test shown in Figure 2b demonstrates the catalyst robustness under operation for 70 h in comethanation conditions, keeping maximum methane selectivity and excellent CO and CO_2_ conversions.

#### 3.1.3 Solo CO Methanation (CO + H_2_)

In contrast to the excellent performance observed in the solo CO_2_ methanation and comethanation, the solo CO methanation (Figure 3) does not result in positive outcomes with the NiO-CeO_2_ (Np) catalyst. While CO methanation onset is quite low around 100 °C, the CO conversion barely reaches the maximum value of 41% at 250 °C and then it decays to 30% at 300 °C. Onward, the CO conversion increases at a moderate pace from 300 to 400 °C until 45% yet does not meet the satisfactory activity. Ni-based catalysts are regarded as deactivating under CO methanation conditions, owing to the accumulation of carbide species on the metal active sites. Alternatively, the volatilization of Ni­(CO)_4_ species is also a proposed potential cause for the sequential activity loss during CO methanation experiments. In the case of the activity in the tested conditions, no severe deactivation was observed for the NiO-CeO_2_ (Np) catalyst as CO conversion was stable despite being low.

**Figure 3:**
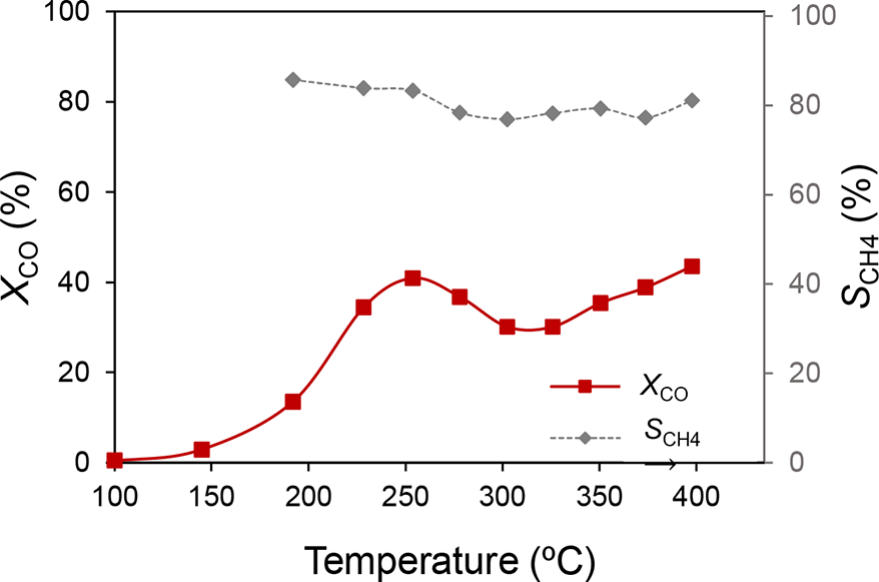
CO conversion profile (*X*_CO_, squares) and CH_4_ selectivity (*S*_CH4_, dashed line) in the fixed-bed activity test in the solo CO methanation with the NiO-CeO_2_ (Np) catalyst.

In relation to the CH_4_ selectivity, the obtained products are a mixture of CH_4_ and CO_2_, rendering an average selectivity of 80% throughout the experiment. The origin of CO_2_ can be attributed to the Boudouard reaction (or CO disproportionation into CO_2_ and C) on the catalyst surface under the CO methanation conditions. In parallel to the Bouduard reaction, since CO is a better reducing agent than H_2_ in ceria-based systems, part of the production of residual CO_2_ can also be attributed to the extensive catalyst reduction by CO even from the reduced resting state after the pretreatment in H_2_. Altogether, CO conversion does not reach relevant values at the experiment temperature range, which means that CO_2_ copresence is necessary to successfully activate CO hydrogenation.

One hypothesis we can propose is that CO_2_ would inhibit CO disproportionation, shifting the thermodynamics toward a selective CO methanation when both are co-fed. However, there were no signs of C deposited on the catalyst after the long-term CO methanation operation, as checked by thermogravimetry. The other hypothesis, based on the catalyst's exhaustive reduction by CO as the cause of the CO_2_ formation, is not conclusive, as in steady state, CO_2_ production should cease at each temperature, and gas levels after 30 min stabilization show continuous CO_2_ release during the experiment after 200 °C (please see Figure S1). Thus, either the system is not in equilibrium at each temperature, or the source of the CO_2_ formed is another. A plausible mechanism for the continuous CO_2_ formation could be the competitive CO adsorption in the form of mono and bidentate carbonates on ceria,^27^ which are stabilized on the reduced ceria surface and eventually slowly decompose into CO_2_ + H_2_O by the effect of H_2_. However, we cannot rule out the mixture of ongoing side reactions in the reactor environment. Namely, the endothermic reverse water gas shift (rWGS) reaction (CO_2_ + H_2_ ↔ CO + H_2_O), which would be activated at higher temperatures than CO methanation, rendering CO and H_2_O from the freshly formed CO_2_ and H_2_. Furthermore, the potential equilibrium in the reactor between the rWGS and its opposite WGS reaction could balance the product yield as influenced by their dependence on temperature along the reaction cycle.

To shed some light on the CO_*x*_ methanation mechanism, in situ DRIFTS experiments were performed under different atmospheres.

### 3.2 In Situ DRIFTS Experiments

In situ DRIFTS experiments were carried out to obtain additional information about the reaction mechanism. Figure 4 shows the spectra at selected regions of interest recorded at different atmospheres under steady state reaction conditions at 300 °C. A background recorded at the same temperature in a N_2_ flow for each sample was subtracted in all cases. Between the atmosphere switch, the materials were reduced in H_2_ at 300 °C as a restoring treatment of the catalyst surfaces.

**Figure 4:**
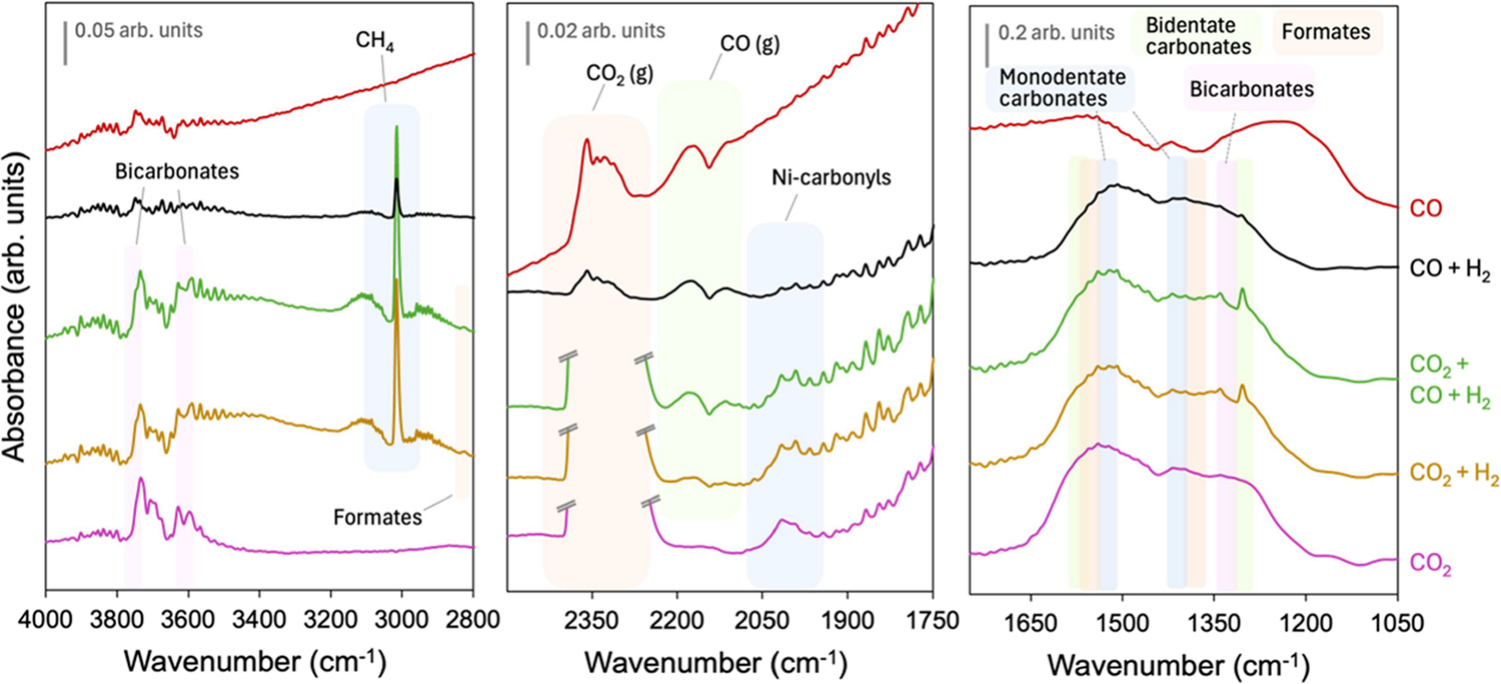
In situ DRIFTS experiments in different atmospheres at 300 °C.

The selected regions depict the surface intermediates formed upon exposure in the different atmospheres. The intense band around 2350 cm^–1^ belongs to CO_2_ in the gas phase, which has been cut in the spectra where CO_2_ is fed for the sake of clarity. As shown in Figure 4, CO_2_ gas is produced in a CO + H_2_ atmosphere, and especially in a pure CO atmosphere. The presence of CO_2_ suggests the occurrence of the Bouduard, or CO disproportionation, reaction (2CO → CO_2_ + C), which tends to hamper the activity of the catalyst. The higher CO_2_ band area in this spectrum, next to the signal drift of the spectra recorded under CO, reveals that the catalyst is undergoing a further reductive process from the resting state, as the background spectrum was taken under H_2_. Thus, in CO + H_2_ conditions, part of the formed carbonyls is stepwise hydrogenated to form methane, while in pure CO, it accumulates, yielding a reduced surface. Catalyst extensive reduction, evidenced by the data drift (mismatch between spectrum and background in H_2_), occurs only in this specific case (CO). Under all the conditions, the catalyst operates without forming coke or irreversible carbonyl species, in analogue performance as that reported in the methane dry reforming process.^14^

CO gas typically presents a roto-vibrational band split into two branches at 2114 and 2170 cm^–1^, which is discernible in the CO-containing atmospheres. Interestingly, a small CO gas band can be identified in the CO_2_ + H_2_ experiment, indicating a residual formation of CO under CO_2_ methanation. However, no CO is released in the CO_2_ methanation fixed-bed activity tests, though, which can be due to the subsequent fast hydrogenation of CO in the reaction conditions. This output could suggest that the principal mechanism of CO_2_ methanation with the NiO-CeO_2_ (Np) catalyst presents CO as an intermediate, either by the straight dissociative mechanism of CO_2_ or through the formate route in the associative mechanism. The weak Ni-carbonyl bands observed in the range 1990–2050 cm^–1^ further confirm the transient CO formation, even under pure CO_2_. CO_2_ is well-known to oxidize the ceria catalyst surface, occupying the oxygen vacancies, rendering CO, which can form carbonyls in the nearby Ni sites. Conversely, the carbonyl band in CO + H_2_ appears much smaller than that in the other conditions, which may be due to a fast hydrogenation of carbonyls once formed. Unfortunately, the noticeable data drift in the CO-recorded spectrum does not enable the resolution of the specific nickel carbonyl species formed.

The 3015 cm^–1^ band is assigned to CH_4_ gas, the CO_*x*_ methanation product, with characteristic overtones. C–H stretching bands of formate species are depicted at 2826 cm^–1^, in the CO_2_ + H_2_ and CO + CO_2_ + H_2_ spectra, while these are not detectable in the CO + H_2_ conditions at 300 °C. The presence of formates could suggest that CO_2_ methanation occurs through a mixture of both dissociative (via CO + OH hydrogenation) and direct hydrogenation (via formate) mechanisms. Above 3500 cm^–1^, the observed bands are ascribed to O–H bonds from isolated hydroxyls on ceria and hydrogen carbonates (3620 and 3734 cm^–1^). Below 1700 cm^–1^, CO stretching modes characteristic of monodentate carbonates (1060, 1400, and 1525 cm^–1^), bidentate carbonates (1303 and 1565 cm^–1^), formates (1373 and 1539 cm^–1^), and bicarbonates (1342 and 1640 cm^–1^) are depicted.^16,28–32^

When the catalyst is exposed to CO_2_, the spectral bands that appear are consistent with the presence of monodentate and bicarbonate species. These species are formed upon CO_2_ chemisorption on ceria, where they are stabilized in the absence of hydrogen. In the CO_2_ methanation mixture (CO_2_ + H_2_), the catalyst shows the same broad bands as with CO_2_ but also an additional sharp band corresponding to bidentate carbonates (1303 cm^–1^). Bidentate carbonates are formed by CO_2_ adsorption on 1 O site and 1 O vacancy on ceria, being the latter generated by the reducing effect of H_2_. There is no clear evidence of the potential implication of these bidentate carbonates as intermediates in the CO_2_ methanation mechanism, but their formation is a sign of CO_2_ activation.

In the simultaneous CO and CO_2_ feeding (CO + CO_2_ + H_2_), the observed surface intermediates are the same as in CO_2_ + H_2_, which suggests the mechanism proceeds the same way: (i) CO_2_ splitting into CO, or formate decomposition to CO; (ii) then stepwise hydrogenation of CO to methane. Thus, CO methanation within the comethanation atmosphere presents a shorter path and a lower onset. However, when the catalyst is in under the CO methanation mixture (CO + H_2_), average CO bands remain unaltered, except for the bidentate carbonate band, which is weakened. As the CO conversion profile in the CO methanation experiment (Figure 3) revealed irregular behavior throughout the reaction cycle, we conducted in situ DRIFTS analyses at different temperatures under the mixture of CO + H_2_ in 1:9 CO:H_2_ stoichiometry.

The spectra shown in Figure 5 shed light on the behavior of the catalyst along the CO methanation reaction cycle. At low temperatures (<250 °C), the catalyst performs via a formate mechanism, which is formed by CO + OH coadsorption on the ceria surface (bands at 1365, 2830, 2934 cm^–1^). Additionally, Ni carbonyl bands are clearly discerned (2030, 1915, and 1882 cm^–1^), in agreement with the strong CO binding affinity on the metal sites. Initially, in the form of linear (2030 cm^–1^) and 2-fold bridging (1915 cm^–1^) Ni carbonyls at 200 °C, then at 250 °C as a 3-fold bridging (1882 cm^–1^) form. On the other hand, bidentate carbonates (1300, 1568 cm^–1^) are identified, potentially by the readsorption of CO_2_ formed upon the interaction of CO with the catalyst ceria surface, or by the CO stabilization on the ceria surface as carbonates.^27^

**Figure 5:**
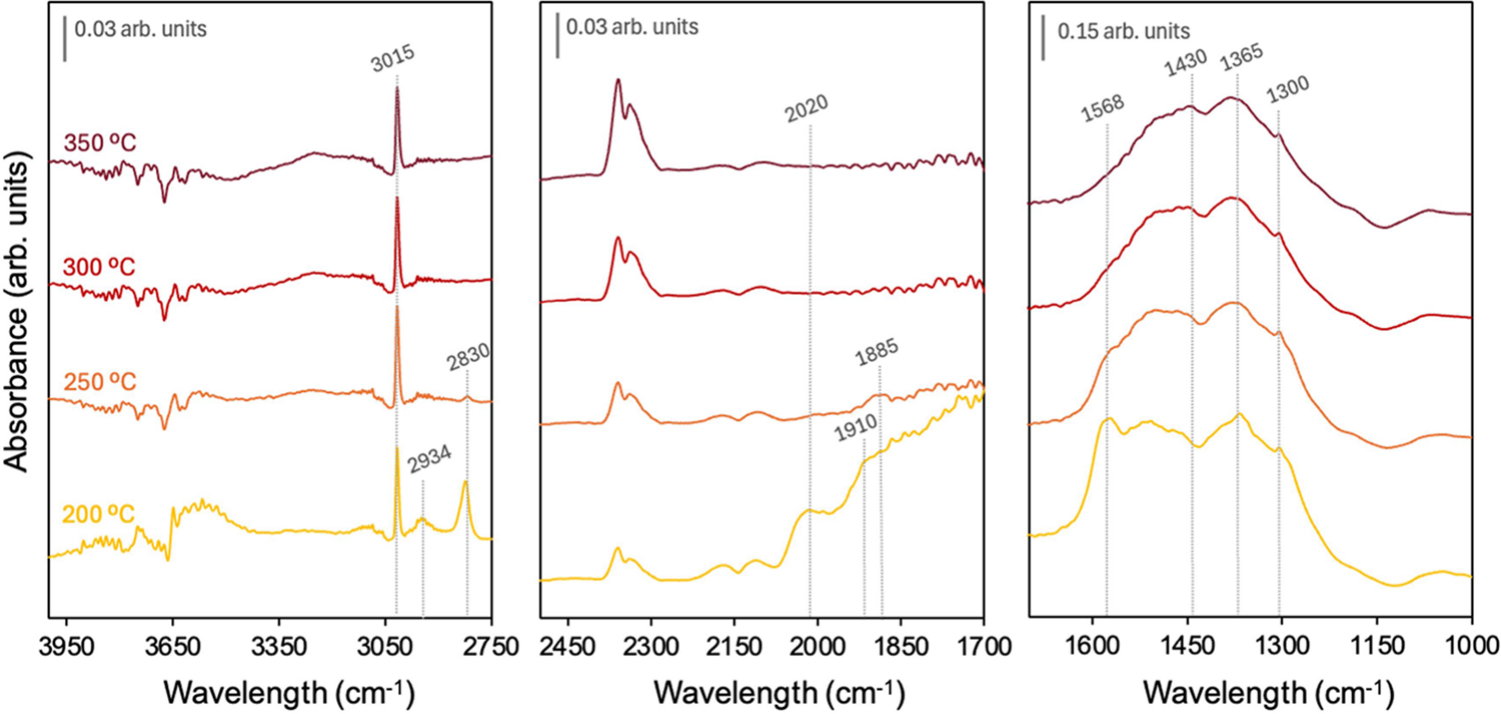
In situ DRIFTS experiments under a CO + H_2_ atmosphere at different temperatures.

As the temperature is raised to 250 °C, formates are consumed, in agreement with the activity increase in this range, suggesting that CO methanation proceeds initially through an associative mechanism. However, beyond 250 °C, the catalyst suffers from activity loss, which may be due to the destabilization of carbonyl species as the CO partial pressure of the CO decreases by the effect of the CO conversion. Thus, while formates are being depleted, carbonates are preserved during the reaction, even in the low activity regime. In agreement with the CO + H_2_ spectrum at 300 °C shown in Figure 4, the absence of nickel carbonyls and formates on the catalyst surface beyond a certain temperature threshold could suggest that CO reduces the catalyst surface, oxidizing itself to CO_2_, which forms carbonates to be later hydrogenated. Thus, the catalyst presents an oxidative/reductive cycling balanced by the reduction by CO and CO_2_ reoxidation that hampers the overall CO methanation process. This behavior is different than the one observed when CO_2_ is cofed next to CO under the comethanation mixture, since CO_2_ already saturates the catalyst surface with carbonates and CO is pushed to its hydrogenation.

The proposed mechanism for CO methanation agrees with the catalyst behavior observed in the in situ DRIFTS for the comethanation mixture (Figure S2), where formates are discerned until 300 °C, besides the sharp band of bidentate carbonates.

Lastly, Figure S3 shows the DRIFT spectra of the catalyst surface after flushing with H_2_ for a long time after each of the atmosphere exposures at 300 °C. As seen, the broad carbonate band is resistant to the depletion by H_2_ reduction except for the catalyst previously subjected to CO, which remains in its reduced state. Bidentate carbonate sharp bands (1303 cm^–1^) are not present, nor nickel carbonyls (1990 to 2050 cm^–1^) in any case, regardless of the previous conditions.

Altogether, these in situ experiments indicate that the surface of the NiO-CeO_2_ (Np) catalyst conducts CO hydrogenation through the same mechanism as CO_2_ hydrogenation in the comethanation mixture. Furthermore, these findings suggest that the CO hydrogenation mechanism generates stable carbonates, as in the CO_2_-fed mixtures.

### 3.3 Isotopic Pulse Experiments In Situ DRIFTS-MS

Additional information about the reaction mechanism was obtained from isotopic ^13^C^18^O_2_ (49) and ^12^C^18^O (30) pulse experiments performed at 350 °C with the NiO-CeO_2_ (Np) catalyst monitored by mass spectrometry and fast-scan DRIFT spectroscopy under different conditions: CO_2_ (49) pulses under H_2_ flow (Figure 6a), CO (30) pulses under H_2_ flow (Figure 6b) and CO (30) pulses under CO_2_ methanation mixture (CO_2_ (44) + H_2_) (Figure 6c).

**Figure 6:**
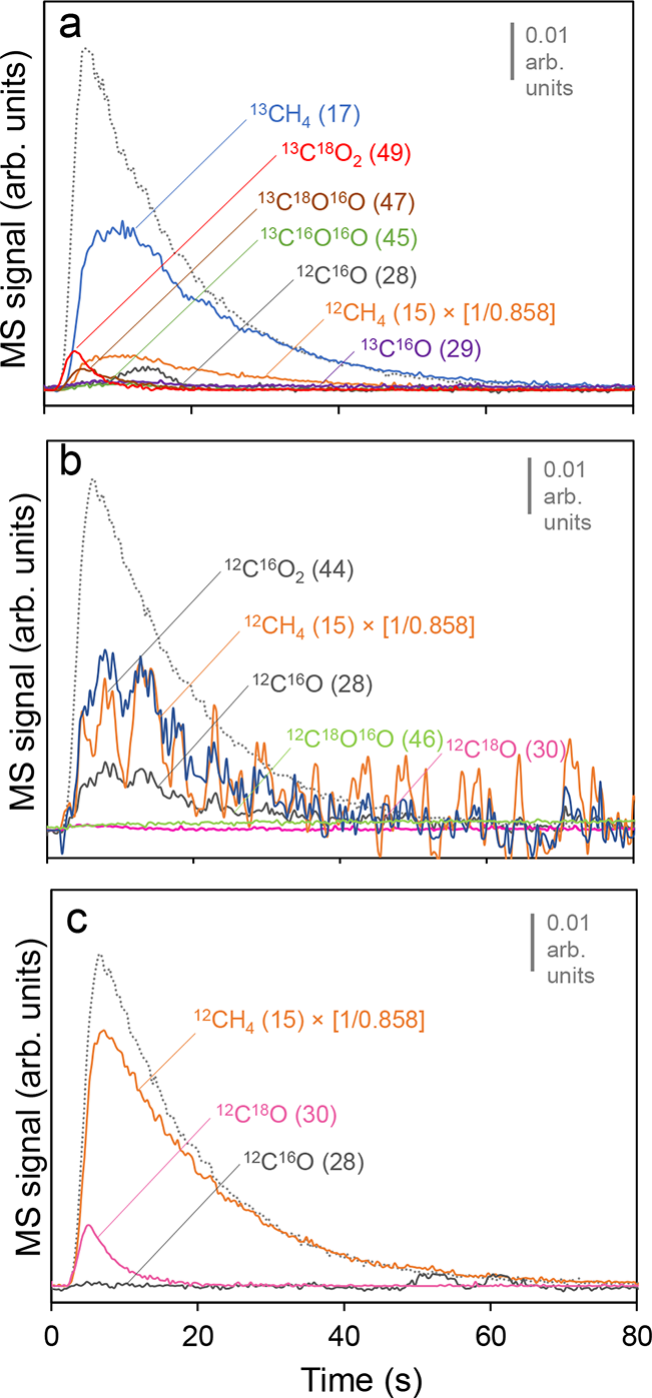
MS signals of released products after gas pulse experiments at 350 °C. (a) ^13^C^18^O_2_ (49) pulse under H_2_ flow; (b) ^12^C^18^O (30) under methanation gas mixture (CO_2_ + H_2_); (c) ^12^C^18^O (30) under H_2_ flow. Ar (40) pulses (dotted lines) were used for calibration, and reproducibility analysis are included as a reference for the residence time.

Isotopic pulses at 350 °C, shown in Figure 6a–c, depict information about the catalyst mechanism at an advanced degree of the CO_*x*_ methanation reactions. Figure 6a (CO_2_ methanation conditions) shows that part of the pulsed ^13^C^18^O_2_ (49) gas is released without reacting, fitting with the thermodynamic constraints of the system. However, the main species detected are methane isotopologues: ^13^CH_4_ (17) and ^12^CH_4_ (16), in agreement with the excellent activity of the NiO-CeO_2_ (Np) catalyst. Since ^13^CH_4_ (17) fragmentation leads to ^13^CH_3_ (16) contributing to ^12^CH_4_ (16) signal, *m*/*Q* = 16 cannot be used to monitor nonisotopic methane. Therefore, ^12^CH_4_ was followed and determined by means of the ^12^CH_3_ (15) fragmentation signal, considering the relative sensitivity factor between 15/16 *m*/*Q* signals = 0.858. The prevalence of ^13^CH_4_ (17) over ^12^CH_4_ (16) suggests that incoming ^13^C^18^O_2_ (49) molecules are stepwise hydrogenated, while a minority of intrinsic carbonaceous species present in the catalyst are also mobilized and hydrogenated upon the pulse as ^12^CH_4_ (16), in concordance with the conclusions of the DRIFTS experiments. Additionally, scrambled ^13^C^18^O^16^O (47) and ^13^C^16^O_2_ (45) and ^13^C^16^O (29) species were marginally detected, evidence that oxygen exchange is fast in the catalyst. The detection of ^13^C^16^O (29) after the pulse in Figure 6a is consistent with the formation of nickel carbonyls. Finally, ^12^C^16^O (28) was released seconds delayed after the pulse, suggesting that the pulsed CO_2_ spurs the intrinsic carbonates' partial reduction, in line with the ^12^CH_4_ (16) production. Carbon monoxide is not detected in the fixed-bed catalytic tests (Figure 1b), and this discrepancy with the pulse experiments can be attributed to the different experimental conditions, such as the CO_2_:H_2_ transient ratio.

The ^12^C^18^O (30) pulse experiment under a CO_2_ methanation atmosphere (Figure 6b) reveals the near-complete consumption of ^12^C^18^O (30), in agreement with the catalytic tests conducted in the comethanation conditions. Besides, in accordance with the DRIFTS experiments, it is demonstrated that CO species are easily hydrogenated when CO_2_ is copresent. In fact, the ^12^C^18^O (30) pulse leads to the release of ^12^C^16^O^16^O (44), as the methanation equilibrium is shifted by the entrance of CO. The noisy signals of the released products in this pulse are consequences of the high MS signals from the background conditions (CO_2_ + H_2_), which are subtracted from each evolved MS signal baseline, as at this temperature, CO_2_ methanation is occurring at an advanced extent. On the contrary, nonisotopic ^12^C^16^O (28) is also detected, potentially produced by oxygen exchange of the incoming ^12^C^18^O (30) molecules, but more likely by the ^12^C^16^O^16^O (44) fragmentation, as it is fed in large amounts in the experimental conditions.

Lastly, the ^12^C^18^O (30) pulse experiment (Figure 6c) shows that the prevailing process is the methanation of the incoming ^12^C^18^O (30) molecules to ^12^CH_4_ (16) although part of the ^12^C^18^O (30) pulse residual is released without reacting. Thus, the overall yield is superior to that observed in the CO methanation catalytic activity tests, as CO conversion is not high in the 200–350 °C range. Additionally, CO_2_ species are not released in the transient isotopic experiments, in contrast with the catalytic tests. The discrepancies can be ascribed to the different CO:H_2_ ratios in the transient pulse experiments with the fixed-bed tests. We attribute such differences to the much greater H_2_:CO ratio of the pulse experiments. Interestingly, ^12^C^16^O (28) was also identified by the mass spectrometer in low quantities, suggesting the oxygen exchange of incoming CO molecules with the ceria lattice.^33^

In relation to the released water species, Figure 7 depicts the *m*/*Q* 18 and 20 signals after each of the pulses. As shown, only after the CO_2_ (49) pulse under H_2_ does it evolve a small nonisotopic water peak, while no water is released under the other pulse conditions. Conversely, no isotopic water (*m*/*Q* = 20) is observed regardless of the pulse, which, together with the greater abundance of nonisotopic CO_2_ and CH_4_ species, suggests that a rapid oxygen exchange occurs in the catalyst, which absorbs part of the inlet isotopic oxygen, refilling catalyst oxygen vacancies.

**Figure 7:**
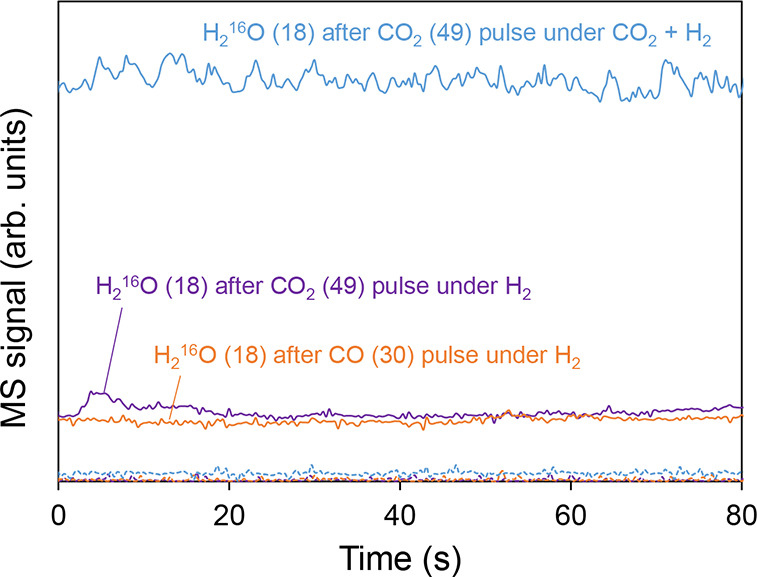
MS signals of released H_2_O products after gas pulse experiments. Solid lines: H_2_^16^O (18); dotted lines: H_2_^18^O (20).

Altogether, the pulse isotopic experiments complemented with the in situ DRIFTS study provide evidence that CO is a reaction intermediate of the NiO-CeO_2_ (Np) catalyzed CO_2_ methanation reaction. CO is not detected in the fixed-bed catalytic experiments, as these are conducted with a stoichiometric CO_2_:H_2_ mixture or with a little excess of H_2_ in the CO:CO_2_:H_2_ and CO:H_2_. Therefore, CO, when produced, is further hydrogenated to methane and not released. On the contrary, pulse experiments are performed in transient conditions with a local excess of CO_2_ or CO during the pulse, and the lack of stoichiometric H_2_ once CO_*x*_ is pulsed enables the CO release.

The isotopic pulse experiments also reveal the absence of ^18^O-containing reaction products, which indicates that the oxygen atoms from the pulsed ^13^C^18^O_2_ (49) are retained on the catalyst, while the reaction products incorporate oxygen from the catalyst (^16^O). A result consistent with the high oxygen anion mobility in the ceria lattice.^33^

Figure 8a reveals that after a ^13^C^18^O_2_ (49) pulse, monodentate and bidentate carbonates are formed as evidenced by their characteristic band region (1200–1550 cm^–1^). With time, the band signals decay as carbonates undergo hydrogenation to form CH_4_. In these conditions, formates and bicarbonates are not observed, likely due to their high relative rate of hydrogenation. Additionally, the IR spectra reveal that a small fraction of ^13^C^18^O_2_ (49) and their isotopic scrambled species are detected within the first seconds after the pulse as red-shifted CO_2_ (g). In the IR spectra of ^12^C^18^O (30) pulsed under the methanation atmosphere (Figure 8b), the bicarbonates and the mono- and bidentate carbonates bands do not show changes when compared to the background of the CO_2_ methanation atmosphere, which is ascribed to the surface saturation of CO_2_-based species. On the other hand, a growth in the CO_2_ (g) band is observed, in agreement with the quantitative pulse analyses. Thus, the CO introduction amid a CO_2_ methanation mixture evolves in the destabilization of the ceria carbonates and their release as CO_2_, while new carbonates are being substituted upon the CO. Lastly, in the ^12^C^18^O (30) pulse (Figure 8c), the absence of CO (g) and carbonyl bands suggests that CO is either directly hydrogenated to methane or retained temporarily in the catalyst as carbonates in transient conditions. Altogether, isotopic pulse experiments reveal that the NiO-CeO_2_ (Np) catalyst presents an active surface for CO_2_, CO, and CO+CO_2_ methanation, which is highly sensitive to changes in local H_2_ partial pressure. CO_2_ methanation takes place through CO as an intermediate, while in comethanation, CO can actively interfere with the ongoing CO_2_ methanation by a preferential adsorption, mobilizing the surface carbonates. Lastly, CO methanation pulsed experiments present high conversion of CO to CH_4_, in contrast to the unsatisfactory outputs from the fixed-bed experiments. Thus, the NiO-CeO_2_ (Np) catalyst presents a high intrinsic CO methanation activity, despite the fact that it presents CO hydrogenation kinetic restrictions in the 1:9 CO:H_2_ experimental ratio.

**Figure 8:**
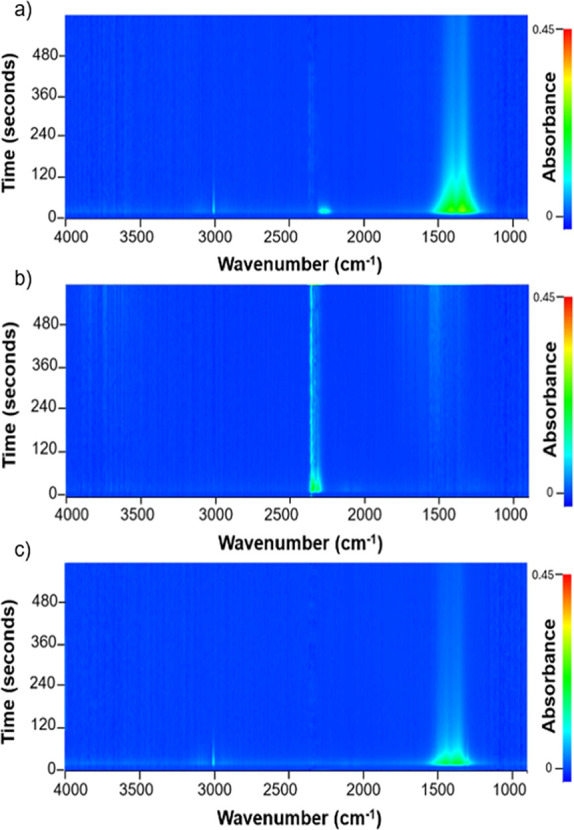
Band density plots in rapid-scan operando DRIFT spectra recorded at 350 °C after ^13^C^18^O_2_ (49) pulse (a); ^12^C^18^O (30) pulse under methanation atmosphere (b); and ^12^C^18^O (30) pulse (c).

### 3.4 NAP-XPS

Near-ambient XPS experiments conducted in a synchrotron provide insights into the dynamic redox changes occurring on the catalyst surface under different reactant conditions. Figure 9a,b show the fitted Ce 3d and Ni 2p_3/2_ XPS region spectra recorded at steady states in different atmospheres, respectively. Ce3*d* XPS region (Figure 8a) was fitted into 10 peaks, corresponding to the overlapping Ce 3d_5/2_ and Ce 3d_3/2_ spin–orbit coupling splits of CeO_2_ and Ce_2_O_3_ oxides.^34–36^ Peaks ascribed to Ce^3+^ surface cations are labeled in Figure 9a, and the quantitative analysis results are depicted in Figure 9c. According to the outputs, the catalyst presents a highly reduced CeO_2_ surface with 55% Ce^3+^ on average. The presence of abundant surface Ce^3+^ relates to a high concentration of oxygen vacancies, which are regarded as CO_2_ activation sites under CO_2_ methanation. On the other hand, the fitted Ni 2p_3/2_ XPS spectra are disclosed in Figure 9b. Four main contributions can be depicted, being the reduced Ni^0^ peak with center at 852.6 eV, oxidized Ni^2+^ peak around 853.7 eV, Ni^2+^ cations in intimate contact with CeO_2_ with lowered electron density by the synergistic redox interplay (Ni^2+^-Ce) presenting its peak around 854.7 eV,^37^ and a higher binding energy peak potentially ascribed to nickel carbonates, nickel hydroxide and other minor species. In the big picture, surface nickel species are mainly present in the form of Ni^0^ in all of the measured conditions, with slight variations. As previously reported, the redox synergism in nickel-ceria catalysts renders the unique feature of active and stable cationic Ni species in intimate contact with CeO_2_, with enhanced capacity to activate CO_2_ molecules.^28^ Thus, complete Ni reduction is not expected, nor even desired, in the NiO-CeO_2_ (Np) catalyst.

**Figure 9:**
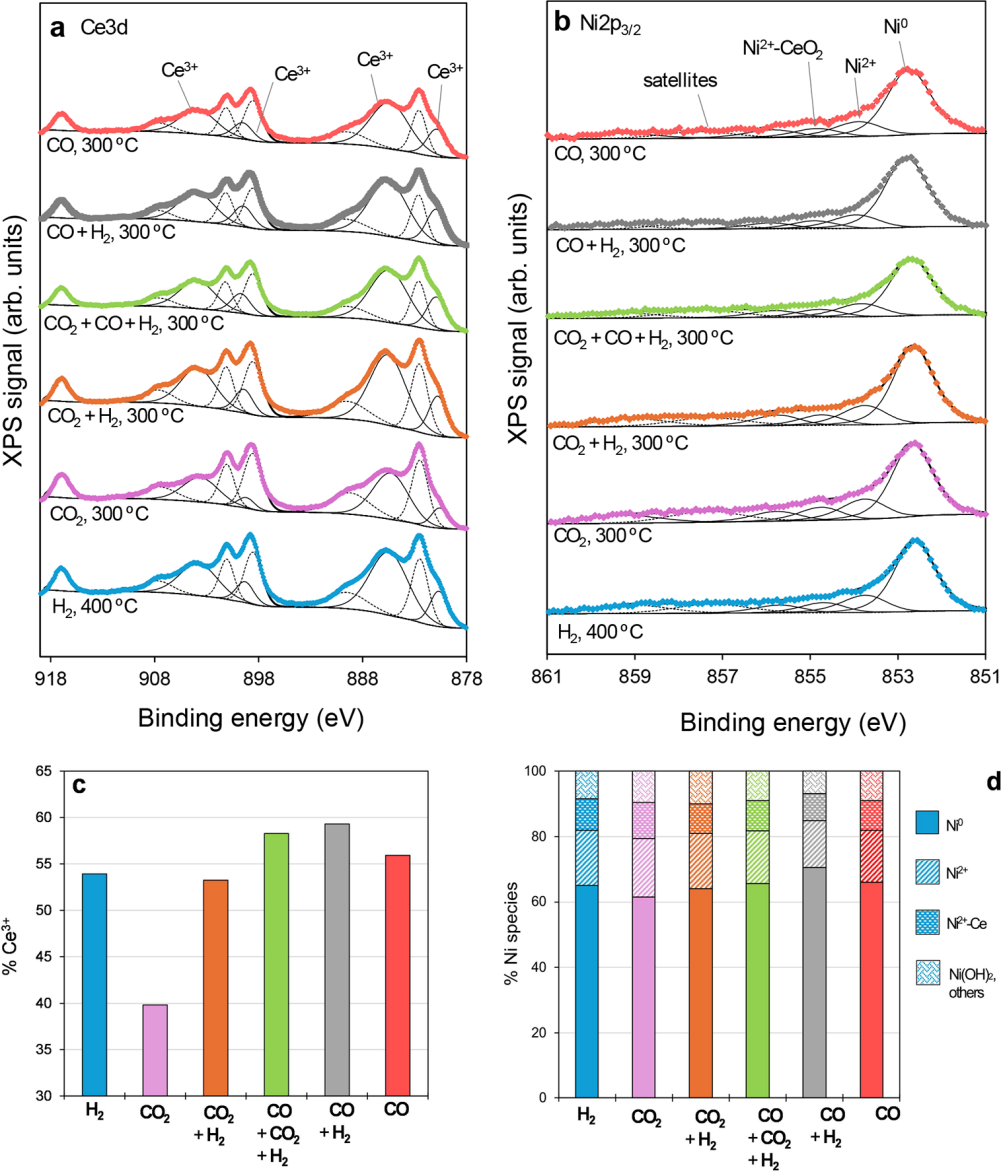
(a) Ce 3d XPS spectra; (b) Ni 2p_3/2_ XPS spectra; (c) quantification of surface reduced Ce^3+^ cations; (d) Ni species distribution in NiO-CeO_2_ (Np) catalyst under different conditions.

Under H_2_ at 400 °C, the ceria surface is reduced by 54% of Ce^3+^, and the nickel species share is 65% Ni^0^, 17% Ni^2+^, 10% Ni^2+^-CeO_2_, and 8% of the miscellaneous high binding energy contribution. This is considered the catalyst resting state, as all reaction cycles are performed after the catalyst pretreatment reduction under these conditions. The cyclability of the redox states was confirmed by recording the XPS spectra under H_2_ between the different reaction steps, as illustrated in Scheme 1. Under a pure CO_2_ atmosphere, ceria is noticeably reoxidized, reducing the %Ce^3+^ up to 40%. On the contrary, nickel species are less sensitive to the reoxidation by CO_2_, scarcely lowering the Ni^0^ fraction to 62%. This means that CO_2_ molecules target mainly the oxygen vacancy sites from ceria, while nickel plays a secondary role in the activation of CO_2_.

Under CO_2_ methanation conditions (CO_2_ + H_2_), the catalyst surface is reduced near the state achieved in pure H_2_, suggesting that the reducing effect of H_2_ prevails over the oxidative impact of CO_2_ on the catalyst. According to the catalytic behavior of the material in the CO_2_ methanation fixed-bed activity tests (Figure 1a), at 300 °C, the maximum CO_2_ conversion limited by the reaction thermodynamics is reached. In the XPS analysis chamber, although differences in the reaction advancement degree at the same temperature are expected, owing to the significant pressure dissimilarities in the experimental setups (1 bar vs 1 One mbar), among other factors. As a result, the lower pressure in the XPS analysis chamber would shift the reaction to higher temperatures, implying that at 300 °C in the XPS measurements, the CO_2_ conversion should be low. Thus, in the early stages of the CO_2_ methanation reaction, the surface of the catalyst remains reduced as in the pretreatment. Under comethanation conditions (CO + CO_2_ + H_2_), CeO_2_ exhibits a higher degree of reduction, increasing the Ce^3+^ ratio to 58%, while barely any changes are observed in the Ni species. Thus, CO favors the reduction of the catalyst in the comethanation mixture. Compared with CO methanation (CO + H_2_), in the absence of CO_2_, Ce^3+^ increases, as does the Ni^0^ share. Since CO + H_2_ is a highly reducing mixture, the NiO-CeO_2_ (Np) catalyst exhibits its maximum reduction state with 59% Ce^3+^ and 71% Ni^0^. Finally, exposing the catalyst to pure CO leads to a less effective catalyst reduction, suggesting that CO interacts more easily with the catalyst surface upon coadsorption of CO and H_2_.

The results from CO_*x*_ methanation NAP-XPS conducted in synchrotron are in good agreement with the mechanism elucidations from isotopic pulse experiments followed by MS and fast-scan DRIFTS. Altogether, CO presents a strong reducing effect on the NiO-CeO_2_ (Np) catalyst in comethanation (CO + CO_2_ + H_2_) and CO methanation (CO + H_2_) mixtures, resulting in the byproduction of CO_2_, which can subsequently be hydrogenated to methane. On the contrary, when fed alone, CO tends to form strongly bound nickel carbonyl groups, while there is a gradual reducing effect on CeO_2_. Thus, the interaction of CO with the catalyst surface presents two competitive behaviors: CO methanation by CO-H_2_ coadsorption and stepwise hydrogenation vs CO reducing effect into the catalyst, rendering CO_2_ as its oxidation product. In the CO + H_2_ experiments (CO methanation), temperature plays a key role in the balance of each of the pathways. Namely, at low temperatures (<250 °C), CO methanation prevails while beyond that temperature threshold, CO methanation activity decays in favor of CO_2_ formation. On the contrary, the scenario is different when the comethanation mixture is fed (CO_2_ + CO + H_2_), since the intrinsic high CO_2_ concentration hampers CO disproportionate, yielding a selective CO_*x*_ methanation. Although CO in the mixture increases the reduction degree of the catalyst as illustrated in NAP-XPS experiments, the copresence of CO_2_ renders a high selectivity toward methane in both CO_2_ and CO reactants.

## 4 Conclusions

In this study, a highly active and selective NiO-CeO_2_ (Np) catalyst based on uniformly sized nanoparticles (7 nm) was synthesized and tested in the CO_*x*_ methanation processes of CO_2_, CO_2_ + CO, and CO. The catalyst performance in both CO_2_ and CO_2_ + CO methanation was superior, reaching the maximum CO_*x*_ conversions constrained by the thermodynamics. Mechanism assessment by means of in situ DRIFTS and pulsed isotopic experiments and NAP-XPS analyses enables us to conclude that CO has a preferential binding affinity to the catalyst surface when compared to CO_2_, initiating its methanation earlier than CO_2_ in the comethanation conditions. In these conditions, despite the fact that the CO_2_ methanation onset shifted to slightly higher temperatures, excellent CO_2_ conversion is achieved at low temperatures (∼300 °C), demonstrating a superior CO_*x*_ methanation activity with robust stability. Conversely, CO methanation conditions (CO + H_2_) presented activity, selectivity, and stability concerns, with low CO conversions throughout the operational temperatures tested. In-depth mechanism analyses have elucidated that in the absence of CO_2_, CO + H_2_ mixtures impact in a truly dissimilar way on the catalyst, with competitive CO hydrogenation and oxidation processes yielding a poor CO methanation performance. This study confirms the NiO-CeO_2_ (Np) catalyst as an outperforming material for CO_*x*_ methanation in CO_2_ + H_2_ and CO_2_ + CO + H_2_ mixtures, while further optimization on the operational conditions for its potential use in CO methanation (CO + H_2_) would be necessary.

## Supplementary Material



## Notes

Methane and carbon dioxide yield during CO methanation reaction in fixed-bed experiments (Figure S1); in situ DRIFTS experiments under CO_2_ + CO + H_2_ atmosphere at different temperatures (Figure S2); and in situ DRIFT spectra recorded in H_2_ flow at 300 °C after exposing the catalyst to the different atmospheres (Figure S3) (PDF)
