# Supplementary material for: Diving into the CO x Methanation Mechanism of CO and CO2 Mixtures Catalyzed by the Nanostructured NiO-CeO2 Catalyst
Source: ACS Catal. 2025 Jul 5;15(14):12192–203. doi: 10.1021/acscatal.5c02682 (PMC12281476; doi:10.1021/acscatal.5c02682)
Supplement: Supplementary file 1 [file cs5c02682_si_001.pdf]

Diving into the CO_x methanation mechanism of CO and CO_2 mixtures catalyzed by nanostructured NiO-CeO_2 catalyst

Iván Martínez-López[1], Juan Bueno-Ferrer[1], Iris Martín-García[1], Esteban Guillén-Bas[1], Arantxa Davó-Quiñonero[1], Virginia Pérez-Dieste[2], Dolores Lozano-Castelló[1], Agustín Bueno López[1]

[1]*Department of Inorganic Chemistry, University of Alicante, Carretera de San Vicente s/n, Alicante, Spain*

[2]*ALBA Synchrotron Light Source, Carrer de la Llum 2-26, 08290 Cerdanyola del Vallès, Barcelona, Spain*

Supporting Information

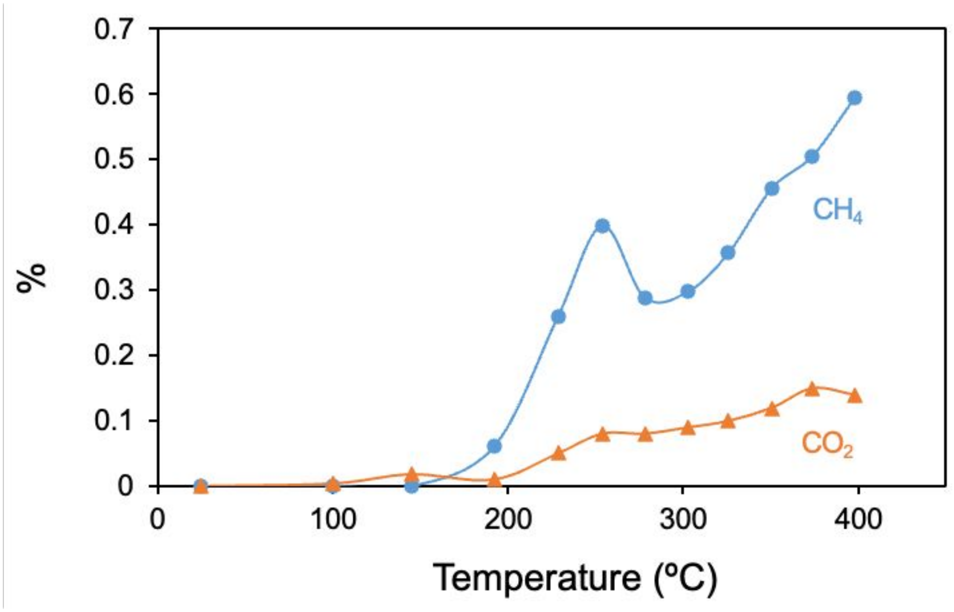

Figure S1. CH_4 (circles) and CO_2 (triangles) yield in the fixed-bed activity test in the solo CO methanation with NiO-CeO_2 (Np) catalyst.

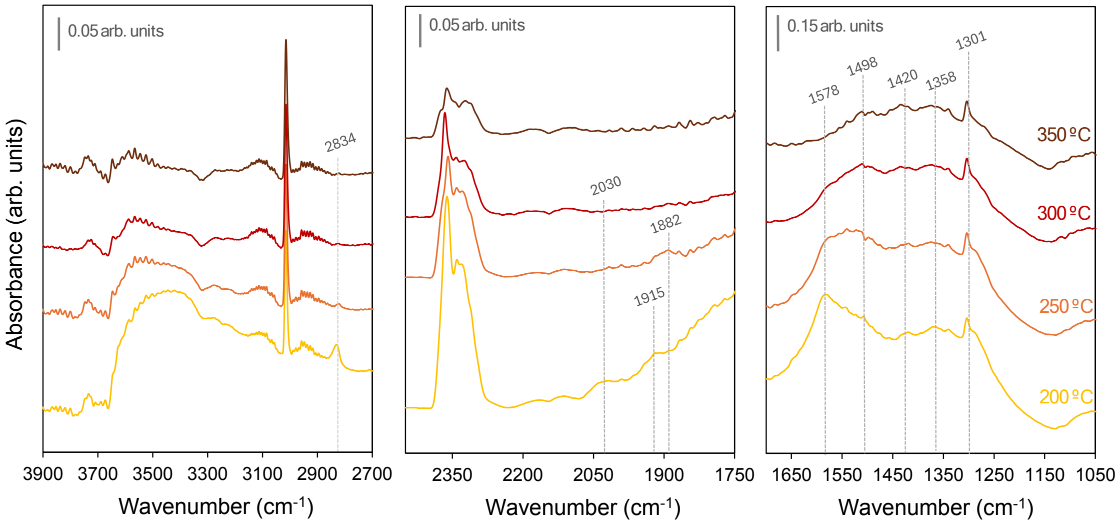

Figure S2. In situ DRIFTS experiments under CO_2 + CO + H_2 atmosphere at different temperatures.

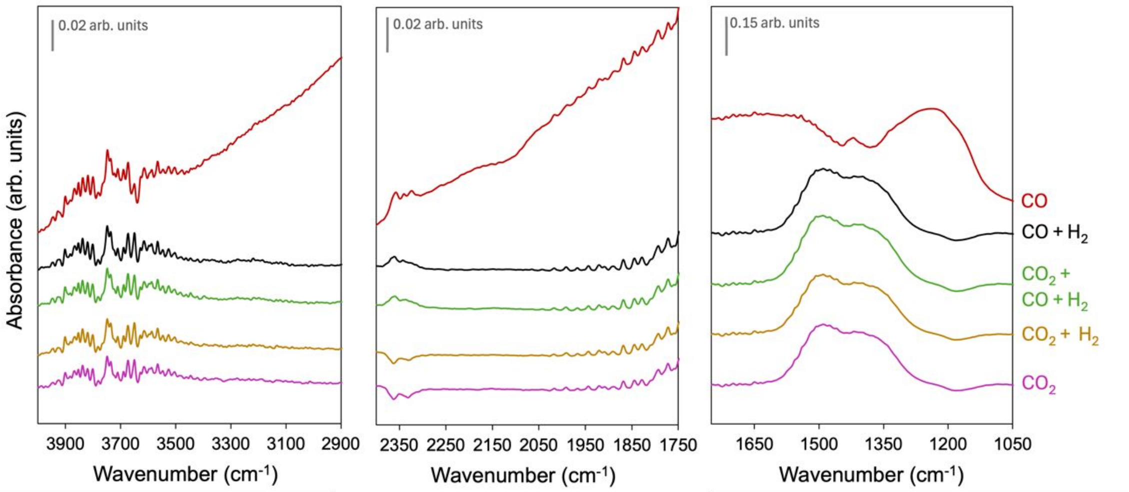

Figure S3. In situ DRIFT spectra recorded in H_2 flow at 300 ºC after exposing the catalyst to the different atmospheres.
